# 3,4-dihydroxyphenyl acetic acid and (+)-epoxydon isolated from marine algae-derived microorganisms induce down regulation of epidermal growth factor activated mitogenic signaling cascade in Hela cells

**DOI:** 10.1186/1475-2867-13-49

**Published:** 2013-05-25

**Authors:** Mi Jeong Jo, Seong Ja Bae, Byeng Wha Son, Chi Yeon Kim, Gun Do Kim

**Affiliations:** 1 Department of Microbiology College of Natural Sciences, Pukyong National University, Busan 608-737, Korea; 2 Department of Chemistry College of Natural Sciences, Pukyong National University, Busan 608-737, Korea; 3 Department of Dermatology School of Medicine, Gyeongsang National University & Hospital, Jinju 660-701, Korea

**Keywords:** EGFR, HeLa, Marine alga, Ishige okamurae, Hypnea saidana, Mitogenic

## Abstract

**Background:**

Epidermal growth factor receptor (EGFR) is a member of the receptor tyrosine kinase (RTK) family. Epidermal growth factor induces its dimerization and stimulates phosphorylation of intracellular tyrosine residues. Phosphorylation of EGFR is studied for cancer therapy because EGFR regulates many cellular processes including cell proliferation, differentiation, and survival. Hence, down-regulation of EGFR kinase activity results in inhibition of signaling cascades amenable for proliferation and progression of cell cycle.

**Methods:**

In the study, we purified 3,4-dihydroxyphenyl acetic acid and (+)-epoxydon from *Aspergillus sp*. isolated from marine brown alga *Ishige okamurae* and *Phoma herbarum* isolated from marine red alga *Hypnea saidana* respectively and determined its anti-tumor activities against HeLa human cervical cancer cells.

**Results:**

Two compounds suppressed EGFR activity *in vitro* with IC_50_ values for 3,4-dihydroxyphenyl acetic acid and (+)-epoxydon were 2.8 and 0.6 μg/mL respectively and reduced the viable numbers of HeLa cells. Immunoblotting analysis exhibited that the compounds induced inhibition of cell growth by causing downregulation of the mitogenic signaling cascade, inactivation of p90RSK, and release of cytochrome c from mitochondria.

**Conclusions:**

Results suggest that decreased expression of active EGFR and EGFR-related downstream molecules by treatment with the compounds may results in the inhibition of cell growth and inducement of apoptosis.

## Background

Epidermal growth factor (EGF) receptor (EGFR) is a type 1 receptor tyrosine kinase or member of the ErbB (HER) receptor family [1]. The EGFR receptor is divided into an extracellular ligand-binding domain, which is an anchor domain that spans the membrane, and an intracellular component that activates tyrosine kinase and induces further downstream signaling [2]. After ligand activation, the members of the family bind to each other, forming homodimers or heterodimers [3]. It has been shown that EGFR is involved in signaling pathways regulating cellular growth, cell cycling, and differentiation [4]. EGFR is overexpressed in various solid tumors including breast, colorectal, ovarian and non-small-cell lung cancer, and excessive EGFR signaling is associated with the development of a wide variety of benign and metastatic tumors [5]. Furthermore, it is reported that when EGFR is overexpressed, it activates the signaling transduction system, and therefore cancer cells grow more aggressively, and with the invasiveness increasing, the transition occur more easily, affecting negative effects to the survival rate [6]. Therefore knowledge of the inhibitory mechanism related to epidermal growth factor (EGF) would assist in searching for targets in cancer therapy [7].

Over the past several decades, many studies on EGFR-targeted therapy in cancer have been performed and numerous targets for anticancer agents have emerged. Especially, monoclonal antibodies (Cetuximab) and tyrosine kinase inhibitors (Gefitinib and Erlotinib) have been developed to inhibit receptor activation [8,9]. Currently, people prefer natural products from the ocean or soil rather than chemical compounds made in a laboratory. In recent years, seaweed extracts have been found to have anti-tumor activities [10], and many researchers have identified algae extracts such as fucoidan and carrageenan which demonstrate anti-tumor effects. The results indicate that the extracts from a wide variety of marine algae could suppress tumor activities and restrain the ability of tumors to grow [11,12]. For this reason, many types of algae extracts have been studied and possible reasons that the extracts inhibit a variety of cancers have been elucidated in the field of cell signaling pathways involving apoptosis, death receptor, and cell cycling [13,14].

Marine fungi were reported having a rich profile of biologically active metabolites [15]. The ecological pressures of a unique marine environment may drive the production of new secondary metabolites by microorganisms. As an example, salinosporamide A was isolated from *Salinispora tropica*, and was found to cause significant proteasome inhibition in clinical trials [16]. Chaetomugilins have been isolated from a strain of *Chaetomium globosum* originally isolated from the marine fish *Mugil cephalus*, and exhibited significant growth inhibition against human cancer cell lines [17].

Although a few studies elicited the bioactivities of metabolites from marine microorganisms, there is no report about the marine microorganisms symbiotic with marine algae. The present study was conducted in order to elucidate the mechanism of the anti-tumorigenic effects of algae derived microorganism extracts, we purified 3,4-dihydroxyphenyl acetic acid from *Aspergillus sp*. on the marine brown alga *Ishige okamurae* and (+)-epoxydon from *Phoma herbarum* on the marine red alga *Hypnea saidana*, respectively. HeLa human cervical epithelial cancer cells were used in the study as highly express EGFR tyrosine kinase on their surface. We studied the inhibitory effects of the compounds on EGF induced phosphorylation of EGFR in HeLa cells. The results of this investigation may provide new insights into the mechanism of tumor suppression and the possibility for applications in tumor prevention and treatment, because control of the activation of EGFR tyrosine kinase has an important role in tumorigenesis [9].

## Results and discussion

### Physiochemical data for 3,4-dihydroxyphenyl acetic acid and (+)-epoxydon from marine algae derived microorganisms

**Figure 1:**
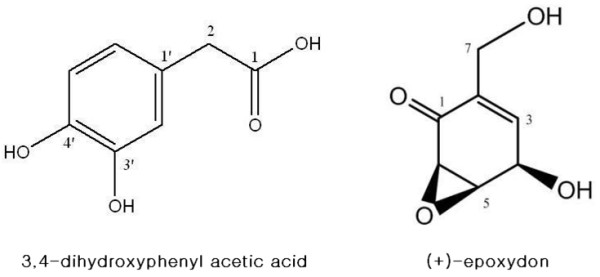
**The effects of the compounds on cell viability.** 3,4-dihydroxyphenyl acetic acid and (+)-epoxydon are isolated from *Aspergillus sp*. derived from marine brown alga *Ishige okamurae* and *Phoma herbarum* isolated from marine red alga *Hypnea saidana*, respectively.

The molecular weight (M.W.) of 3,4-dihydroxyphenyl acetic acid is 168 (C_8_H_8_O_4_) (Figure 1). ^1^H NMR (CDCl_3_, 400 MHz) δ 3.53 (2H, s, H2-2), 6.64 (1H, s, H-2΄), 6.47 (1H, d, J = 7.5 Hz, H-5΄), 6.63 (1H, d, J = 7.5 Hz, H-6΄); ^13^C NMR (DMSO, 100 MHz) δ 173.1 (s, C-1), 40.2 (t, C-2), 125.6 (s, C-1΄), 115.3 (d, C-2΄), 144.0 (s, C-3΄) 145.0 (s, C-4΄), 116.6 (d, C-5΄), 120.0 (d, C-6΄); LREIMS *m*/*z*: 168 [M]^+^(50), 151[M-OH]^+^(29), 123[M-COOH]^+^(100), 107[M-COOH-OH + H]^+^(42), 94(26), 77(42).

The molecular weight (M.W.) of (+)-epoxydon is 156 (C_7_H_8_O_4_) (Figure 1). [α]_D_ + 71.6° (*c* 0.3, MeOH); IR (neat) ν_max_ 3356, 1680, 1400, 1236, 1027, 903, 867 cm^-1^; UV (MeOH) λ_max_ (log ϵ) 203 (3.72), 237 (3.68) nm; CD (MeOH) (Δϵ) 338 (+0.95), 245 (-1.76) nm; ^1^H NMR (400 MHz, DMSO-*d*_6_) δ 6.39 (1H, dddd, *J* = 2.7, 2.6, 2.2, 2.1 Hz, H-3), 4.70 (1H, ddddd, *J* = 6.2, 2.8, 2.8, 2.6, 2.1 Hz, H-4), 5.79 (1H, d, *J* = 6.2 Hz, 4-OH), 3.40 (1H, d, *J* = 4.2 Hz, H-5), 3.76 (1H, ddd, *J* = 4.2, 2.8, 2.7 Hz, H-6), 3.96 (1H, dddd, *J* = 15.2, 5.5, 2.8, 2.2 Hz, H_a_-7), 4.07 (1H, dddd, *J* = 15.2, 5.5, 2.6, 2.1 Hz, H_b_-7), 5.01 (1H, t, *J* = 5.5 Hz, 7-OH); ^13^C NMR (100 MHz, DMSO-*d*_6_) δ 193.9 (s, C-1), 133.8 (s, C-2), 141.4 (d, C-3), 63.7 (d, C-4), 52.9 (d, C-5), 54.0 (d, C-6), 57.3 (t, C-7); CIMS *m*/*z* (rel.int.) 156 [M]^+^ (100), 138 [M-H_2_O]^+^ (7), 122 [M-H_2_O-O]^+^ (2), 110 [M-CO-H_2_O]^+^(3).

### Inhibition of EGFR tyrosine kinase activities

**Figure 2:**
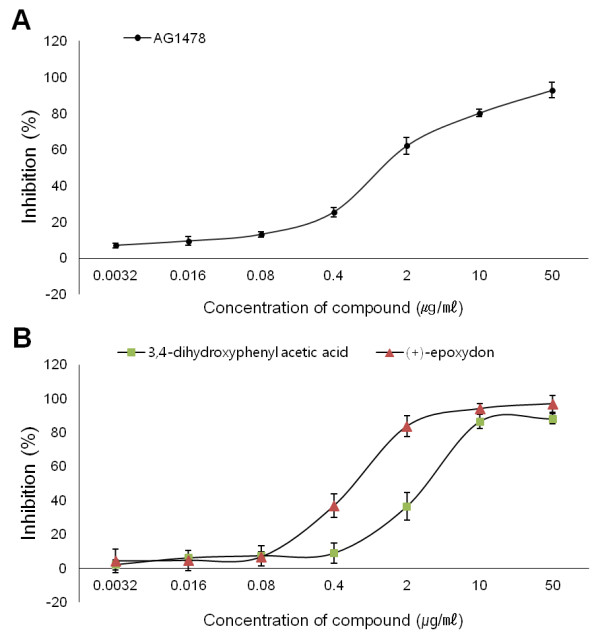
**The inhibitory effects of 3**,**4**-**dihydroxyphenyl acetic acid and (+)-epoxydon on EGFR kinase activity.** The inhibitory effects of the compounds were determined by *in vitro* kinase assay. The assays were performed followed by the procedures described in Materials and Methods. Values shown in the graphs are mean ± SDM and were obtained from three independent experiments.

The inhibitory activity of EGFR tyrosine kinase for two compounds, 3,4-dihydroxyphenyl acetic acid and (+)-epoxydon, were examined *in vitro* using the AlphaScreen P-Tyr-100 assay system (Figure 2). EGF receptor tyrosine kinase assays were performed at the same conditions to compare 3,4-dihydroxyphenyl acetic acid and (+)-epoxydon. The activity of the soluble EGF receptor kinase domain derived from the cytoplasmic portion of the human EGF receptor was tested in the assay. The kinase domain is constitutively active and retains its substrate specificities, kinetic constants and autophosphorylation sites without the requirement for ligand-mediated activation. The assay uses the broad spectrum substrate poly [Glu:Tyr] (4:1), and is based on the binding between the phosphorylated polypeptide and the anti-phosphotyrosine antibody (P-Tyr-100) conjugated to acceptor beads. Shows that both compounds had inhibitory effect on the activity of EGFR in dose-dependent manner, and the half maximal inhibitory concentrations (IC_50_ values) for 3,4-dihydroxyphenyl acetic acid and (+)-epoxydon were 2.8 and 0.6 μg/mL respectively.

### Inhibitory effects of the compounds on EGF-induced cell growth

**Figure 3:**
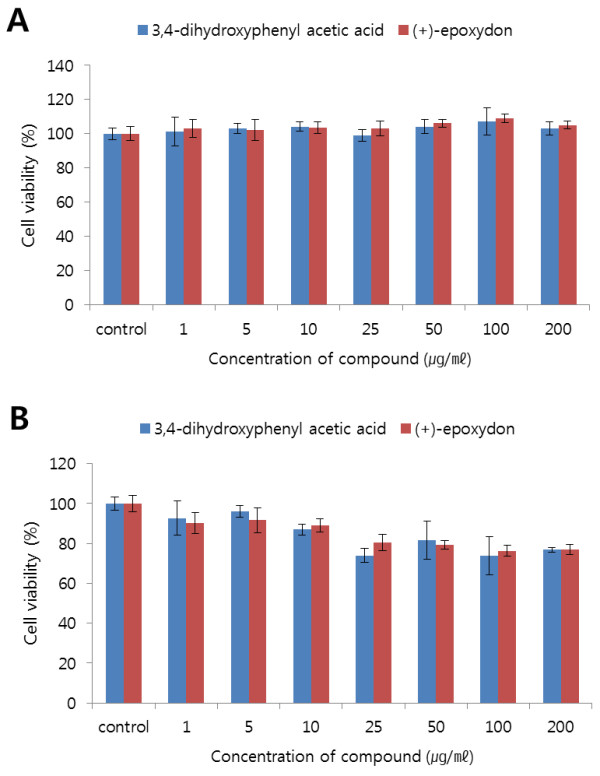
**The effects of the compounds on cell viability.** The anti-proliferation of HeLa and HEK293 cells were examined using WST-1^®^ solution after treatment with each compound and EGF. The HEK293 (**A**) and HeLa (**B**) cells were treated with the indicated concentrations (0, 1, 5, 10, 25, 50, 100 and 200 μg/mL) of each compound for 40 min, and then exposed to EGF (10 ng/mL for 10 min). Values are expressed relative to that of vehicle-treated cells (DMSO), normalized to 100%. Values shown in the graphs are mean ± SEM and were obtained from three independent experiments.

**Figure 4:**
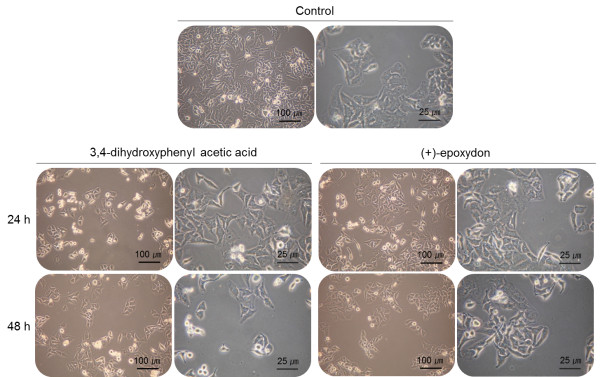
**Morphological changes of HeLa cells by the treatment of compounds.** The cells were treated with each compound (50 μg/mL for 24 or 48 h) and EGF (10 ng/mL for 10 min), except for the control cells treated with EGF only.

Cell viability assays were performed to examine the effects of each compound on cell proliferation. The cells treated with only EGF were defined as a positive control during experiments, while others were pre-treated with each compound at various concentrations prior to treatment with EGF. The results of the cell viability assay showed that two compounds, 3,4-dihydroxyphenyl acetic acid and (+)-epoxydon, did not inhibit cell growth and proliferation in HEK293 (Figure 3A). On the contrary, HeLa cells treated with each compound for 40 min show that the rates of proliferation in HeLa cells are decreased and their inhibition effect was strong on higher concentration (Figure 3B). Additionally, the population of HeLa cells treated with both compounds was reduced during observation under an optical microscope (Figure 4). In comparison to the control cells treated with only EGF, the numbers of cells treated with both compound and EGF were lower than the control numbers. When the cells were exposed to each compound for 48 h, most of cells were dead and floated.

### Activation of EGFR tyrosine kinase by EGF as a positive control

**Figure 5:**
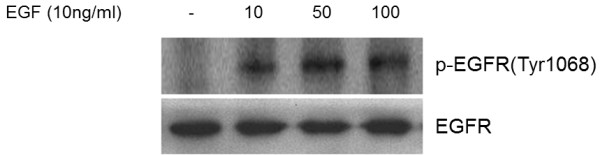
**EGFR (phosphorylation of tyrosine) activation induced by EGF.** Western blot analysis against phosphorylated form of Tyr1068 and non-phosphorylated form of EGFR was performed to examine the effects of various concentrations of EGF (0, 10, 50, 100 ng/ml for 10 min) on the activation of EGFR tyrosine kinase in HeLa cells.

Epidermal growth factor receptor (EGFR) consists of a ligand-binding region and a kinase domain [18]. When the receptor is activated by attachment of an EGF-like ligand on the ligand-binding region, it undergoes a biochemical change to phosphorylate each of the residues on the kinase domain [19]. Especially, phosphorylation of Tyr1068 strongly responds to EGF, and then stimulates mitogenic [Ras/Raf/MEK/extracellular signal-regulated kinase (ERK)] signaling pathways [20]. From Western blot analysis, the expression levels of active EGFR (phospho-EGFR) were stimulated to a greater extent when the cells were treated with EGF (Figure 5). Non-phosphorylated EGFR reflects the total amount of receptor on the cell surface. Thus, it is assumed that the expression level of phosphorylated EGFR depends on treatment with EGF results in its activation even if the amount of receptor exists in the cell is still the same.

### Down-regulation of the activated EGFR by the compounds

EGF plays an important role in the regulation of cell growth, proliferation, and differentiation by binding to its receptor EGFR [21] and stimulating the associated protein tyrosine kinase activity [22]. Epidermal growth factor receptor (HER1) tyrosine kinase is a recognized target for tumor therapy and anti-cancer drugs have been developed to inhibit receptor activation [23]. Researchers have shown that the receptor was suppressed by tyrosine kinase inhibitors (Iressa) [24], monoclonal antibodies such as Cetuximab, and other compounds [23].

**Figure 6:**
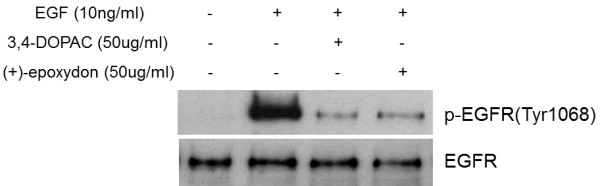
**The inhibitory effects of the compounds on EGF**-**induced activation of EGFR.** Both 3,4-dihydroxyphenyl acetic acid and (+)-epoxydon inhibited EGF-induced phosphorylation of Tyr1068 in EGFR. HeLa cells were treated with each compound (50 μg/mL for 40 min), followed by exposure to EGF (10 ng/mL for 10 min), except for the negative control (untreated).

**Figure 7:**
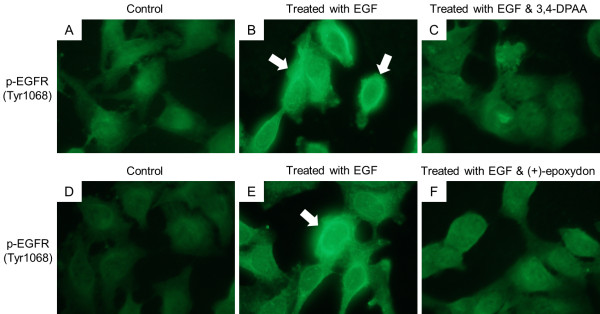
**Immunofluorescence of the expressions of phosphorylated EGFR.** Immunofluorescence was performed as follows by Materials and Methods and the images were captured at 488 nm. In control cells (panels **A** and **D**) and cells treated with EGF (10 ng/mL for 10 min, panels **B** and **E**), EGF induced the expression of active EGFR (white arrows, brightened) but the increased expression levels were not decreased in the cells treated with both EGF and each compound (panels **C** and **F**).

When the cells were treated with each compound before exposure to EGF, the blocking effect was stronger than when not treated (Figure 6). It is believed that EGF binding to the receptor was blocked by those compounds. Results indicated that treatment of the compounds significantly decreased the phosphorylation of EGFR, but not the EGFR total protein level. In addition, the quantitative changes of the phosphorylated EGFR were assayed by immunofluorescence as shown in Figure 7. The specificity of antibodies to their antigen was used to target fluorescent dyes to phosphorylated EGFR within a cell, therefore allowing visualization of the distribution of the receptor molecule through the sample. As a result, the shapes of cells were expressed in green fluorescent light because immunofluorescence makes use of fluorophores to visualize the location of the antibodies and EGFR exists over the entire cell surface. The cells with EGF (Figures 7A and 7D) had more phosphorylated EGFR compared to untreated cells (Figures 7B and 7E) and the active functions were interrupted by the 2 compounds (Figures 7C and 7F).

### Inhibition of EGFR-mediated mitogenic signaling

One of the most important protein kinase cascades activated by tumor promoters, such as EGF, is the mitogen-activated protein kinase (MAPK), induced by the activation of EGFR. Ras is a small guanine-nucleotide binding proteins (G-proteins) cycle between active (GTP-bound) and inactive (GDP-bound) forms [25]. Receptor tyrosine kinases and G-protein-coupled receptors activate Ras, which then stimulates the Raf-MEK-MAPK pathway [26]. Mitogen-activated protein kinases (MAPKs) constitute a widely conserved family of serine/threonine protein kinases involved in many cellular programs such as cell proliferation, differentiation, motility, and death. The p44/42 MAPK (Erk1/2) signaling pathway can be activated in response to a diverse range of extracellular stimuli including mitogens, growth factors, and cytokines [27,28] and is an important target in the diagnosis and treatment of cancer [29]. Upon stimulation, a sequential 3-part protein kinase cascade is initiated, consisting of a MAP kinase kinase kinase (MAPKKK or MAP3K), a MAP kinase kinase (MAPKK or MAP2K), and a MAP kinase (MAPK). MEK1 and MEK2 activate p44 and p42 through phosphorylation of activation loop residues Thr202/Tyr204 and Thr185/Tyr187, respectively. Several downstream targets of p44/42 have been identified, including p90RSK [30] and cytochrome c [31]. Cytochrome c is a well conserved electron-transport protein and is part of the respiratory chain localized to the mitochondrial intermembrane space [32]. Upon apoptotic stimulation, cytochrome c is released from mitochondria, and this event eventually leads to apoptosis [31].

**Figure 8:**
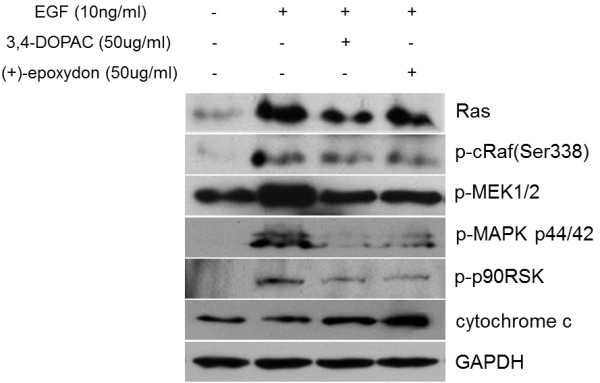
**Inhibition of the activity of signal molecules in mitogenic signaling cascade.** The compounds inhibited EGF-induced phosphorylation of EGFR and its downstream signal molecules in mitogenic signaling. HeLa cells were treated with each compound (50 μg/mL for 40 min) followed by exposure to EGF (10 ng/mL for 10 min) except for the negative control (untreated). Proteins from the cells were resolved by 12% SDS-PAGE and immunoblotted with indicated antibodies. GAPDH was used as an internal loading control.

Therefore, we examined the inhibitory effects of the compounds on the activities of signal molecules in mitogenic signaling cascade. As shown in Figure 8, western blot analysis exhibited that the compounds induced inhibition of cell growth as demonstrated by low expression of signal molecules in the mitogenic signaling cascade and inactivation of p90RSK. Activation of p90RSK by response to growth factor prevents release of mitochondrial cytochrome c and progress of apoptosis. The results suggest that decreased expression levels of active EGFR and EGF-related downstream molecules and the possible involvement of p90RSK by treatment with the compounds may affect the inhibition of cell proliferation and induction of mitochondria mediated apoptosis in Hela cells.

### Relative inhibitory effect of the compounds to Tyrphostin AG 1478 on the activities of EGFR and EGF-related downstream molecules

**Figure 9:**
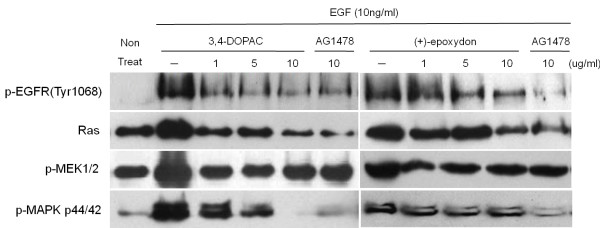
**Relative inhibitory effects of the compounds to Tyrphostin AG 1478 on the activities.** HeLa cells were pre-incubated with different concentrations of each compound or AG 1478 (10 μg/mL) for 40 min and then stimulated with EGF (10 ng/ml for 10 min). Western blot analysis performed with the indicated antibodies.

Tyrphostin AG 1478 (AG 1478) is a tyrosine kinase inhibitor and treatment of cells with AG 1478 can block EGFR activation *in vivo*[33,34]. AG 1478 has been mainly used for laboratory research as an EGFR antagonist. There is increasing evidence to suggest that AG 1478 has anti-tumor activity and significant anti-proliferative effects both *in vitro* and *in vivo*[35]. Western blot analysis exhibited that the compound, 3,4-dihydroxyphenyl acetic acid, could down-regulate the expression of phosphorylated EGFR, Ras, phosphorylated MEK, and MAPK, which may be one of the mechanisms by which AG 1478 suppressed cellular invasion (Figure 9). The inhibitory effect of 3,4-dihydroxyphenyl acetic acid was similar to or even better than AG 1478. In the case of (+)-epoxydon, the inhibitory effect was not as strong as that of AG 1478, but the expression levels of each molecule were effectively decreased by the compound.

## Conclusion

In this study, we demonstrated that 3,4-dihydroxyphenyl acetic acid and (+)-epoxydon from marine algae reduced expression of the mitogenic signaling cascade and EGFR activation, leading to apoptosis in HeLa cells because EGFR has been indicated to be an important target in cancer therapy.

3,4-dihydroxyphenyl acetic acid is a metabolite of the neurotransmitter dopamine, and can be oxidized by hydrogen peroxide, leading to the formation of toxic metabolites which destroy dopamine storage vesicles in the substantia nigra. (+)-epoxydon is a new secondary metabolite from a marine algae derived fungus. In the present study, we purified 3,4-dihydroxyphenyl acetic acid and (+)-epoxydon from *Aspergillus sp*. isolated from marine brown alga *Ishige okamurae* and *Phoma herbarum* isolated from marine red alga *Hypnea saidana* respectively and determined its anti-tumor activity against HeLa human cervical cancer cells.

**Figure 10:**
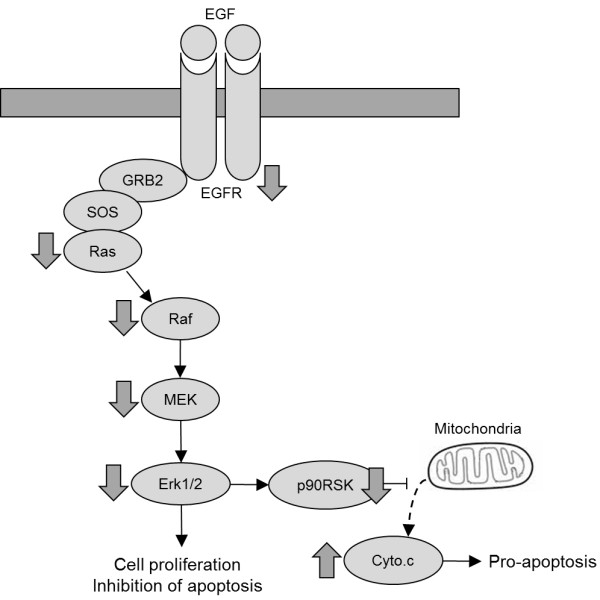
**Proposed signaling cascades of the compounds on EGF-induced p44/42 MAPK (Erk1/2) pathways.** The compounds inhibited the activities of signal molecules in MAPK pathways and increased release of cytochrome c from mitochondria. It may results in inhibition of cell proliferation and apoptosis in HeLa cells.

We investigated the antiproliferation effect of two compounds on HeLa cells. Cell viability assay showed that two compounds, 3,4-dihydroxyphenyl acetic acid and (+)-epoxydon, inhibited cell growth and proliferation at 24 h with IC_50_ at 21.4 μg/mL and 23.8 μg/mL, respectively. The results indicated that two compounds from marine algae derived microorganisms blocked EGF-induced phosphorylation of EGFR, suggesting that both compounds may bind with EGF and prevent the binding of EGF to EGFR. Especially, 3,4-dihydroxyphenyl acetic acid than (+)-epoxydon down-regulated the expressions of phosphorylated EGFR, Ras, phosphorylated MEK ,and MAPK to the same degree as AG 1478, which is also a tyrosine kinase inhibitor. The compounds also blocked the phosphorylation of Ras, Raf, MEK, MAPK and p90RSK-induced cell growth and proliferation. In contrast, the release of cytochrome c, which results in apoptosis, was increased by the compounds (Figure 10).

In conclusion, this study demonstrated that the compounds effectively inhibited proliferation and invasion of HeLa cells and suggests that EGFR may be a potential therapeutic agent for cervical cancer.

## Methods

### Isolation for 3,4-dihydroxyphenyl acetic acid and (+)-epoxydon from marine algae derived microorganisms

The compound, 3,4-dihydroxyphenyl acetic acid (DOPAC), was isolated from the surface fungus of the marine brown alga *Ishige okamurae* collected at Uljin, Gyeongbuk province and Geomoon Island, JeonNam province in South Korea. The fungus was then identified as *Aspergillus sp*. on the basis of morphological evaluation and 18S rRNA analysis (SolGent, Daejeon, South Korea) (similarity 99%). The fungus was cultured (10 L for 4 weeks (static) at 29°C in a SWS medium consisting of soytone (0.1%), soluble starch (1.0%), and seawater (100%). The resulting broth and mycelia were extracted separately with EtOAc (ethyl acetate) and CH_2_Cl_2_-MeOH (methanol) (1:1) to afford the broth extract (430 mg) and the mycelium extract (1.1 g), respectively. The broth extract showed a radical (DPPH) scavenging activity with an IC_50_ value of 1.1 μg/mL, however, the mycelium extract was inactive. Therefore, broth extracts were subjected to column chromatography on silica gel (n-hexane/EtOAc), and then octadesyl silica (ODS) gel (H_2_O/MeOH) to provide 5 fractions. Further purification of fraction-4 containing 3,4-dihydroxyphenyl acetic acid by recycling HPLC, followed by HPLC (C18 Apollo, MeOH-H_2_O = 3:2), yielded 3,4-dihydroxyphenyl acetic acid (3.3 mg).

The other compound, (+)-epoxydon, was isolated from the surface fungus of the marine red alga *Hypnea saidana* collected in Tongnyeong and Yokjee Island, GyeongNam province in South Korea, and then identified as *Phoma herbarum* on the basis of morphological evaluation and 18S rRNA analysis (SolGent, Daejeon, South Korea) (similarity, 99%). The culture broth and mycelia were separated, and the broth (10 L) was extracted with ethyl acetate to provide a crude extract (640 mg) which was subjected to silica gel flash chromatography and eluted with n-hexane/EtOAc (5:1), n-hexane/EtOAc (1:1), n-hexane/EtOAc (1:5), n-hexane/EtOAc (1:10), and finally with EtOAc. The collections (30 mL each) were combined on the basis of their TLC profiles to yield 5 major fractions. Medium pressure liquid chromatography (MPLC) of fraction-3 on ODS by elution with MeOH yielded crude (+)-epoxydon (9.0 mg). The isolated crude (+)-epoxydon was further purified by HPLC (YMC ODS-A, MeOH) utilizing a 30 min gradient program of 50% to 100% MeOH in H_2_O to yield (+)-epoxydon (5.0 mg).

### In vitro assay for EGF receptor tyrosine kinase

The substrate (poly [Glu:Tyr] (4:1)) and the AlphaScreen® P-Tyr-100 assay kit (PerkinElmer Inc., Waltham, MA, USA) composed of Donor-streptavidin and Acceptor-P-Tyr-100 beads were used for the EGF receptor tyrosine kinase assay. EGFR enzyme purified from human carcinoma A431 cells was purchased from Sigma-Aldrich. The kinase reactions were performed in a mixture of EGFR enzyme, ATP, and biotinylated poly [Glu:Tyr] (4:1) in a kinase reaction buffer (50 mM Tris (pH 7.5), 5 mM MgCl_2_, 5 mM MnCl_2_, 2 mM DTT, and 0.01% Tween-20). The mixture was incubated for 1 h at room temperature (RT) and then quenched by addition of detection buffer containing EDTA, Donor-Streptavidin, and Acceptor-P-tyr-100 beads. After further incubation for 1 h at RT, the intrinsic kinetic activities were detected as an AlphaScreen® signal using a Fusion-Alpha microplate analyzer (PerkinElmer Inc.).

### Cell culture and treatment

Human cervical cancer HeLa cells and human embryonic kidney HEK293 (American Type Culture Collection, Manassas, VA, USA) cells were grown in Dulbecco's Modified Eagle's Medium (DMEM) with high-glucose (HyClone Laboratories, Logan, UT, USA), supplemented with 10% heat-inactivated fetal bovine serum (HyClone Laboratories) and penicillin-streptomycin (100 μg/mL penicillin, and 100 units/mL streptomycin) (PAA Laboratories GmbH, PA, Austria) at 37°C with 5% CO_2_. Human epidermal growth factor (Sigma-Aldrich, St Louis, MO, USA) was dissolved in media and treated at a final concentration of 10 ng/mL. Each compound was reconstituted with DMSO to 10 mg/mL and added to the culture media to the final concentration specified in the test. The same concentration of DMSO was added to the control dishes. HeLa cells were treated with 50 μg/mL of each compound for 40 min and then treated with 10 ng/mL of EGF for 10 min, except for control cells.

### Cell viability assay

To estimate the effects of the compounds on cell viability, HeLa and HEK293 cells were seeded (1 × 10^4^ cells/mL) in 96-well plates in 100 μL of DMEM-10% FBS and cultured for 24 h at 37°C in a 5% CO_2_ incubator. After incubation, the HeLa and HEK293 cells were treated with two compounds (0, 1, 5, 10, 25, 50, 100 and 200 μg/mL) for 40 min, and then exposed to EGF (10 ng/mL) for 10 min. After treatment, 10 μL of EZ-Cytox Cell Viability Assay solution WST-1® (Daeil Lab Service, Jong-No, Korea) was added to each well, and the cells were then incubated for 3 h at 37°C in a 5% CO_2_. Absorbance was measured at 460 nm with an ELISA reader (Molecular Devices, Sunnyvale, CA, USA).

### Protein extraction and western blotting

HeLa cells were cultured with each compound for 40 min and then treated with 100 ng/mL of EGF for 10 min. The cells were then washed twice with cold-PBS, harvested and lysed with lysis buffer [50 mM Tris-Cl (pH 7.5), 150 mM NaCl, 1 mM DTT, 0.5% NP-40, 1% Triton X-100, 1% deoxycholate, 0.1% SDS containing proteinase inhibitors (PMSF, EDTA, Aprotinin, Leupeptin, Prostatin A, Intron Biotechnology, Gyeonggi, Korea)]. The lysates were shaken on ice 6 times every 5 min and centrifuged at 14,000 rpm for 20 min at 4°C. Using bovine serum albumin (BSA) as a standard, the Protein Quantification Kit (CBB solution®) (Dojindo Molecular Technologies, Rockville, MD, USA) was used for determining the concentration of whole cell lysates. Each protein was resolved by 12% sodium dodecyl sulfate-polyacrylamide gel (SDS-PAGE) and then transferred onto nitrocellulose membranes (PALL Life Sciences, Pensacola, MI, USA). The membranes were blocked with phosphate buffered saline-Tween-20 (PBST: 135 mM sodium chloride, 2.7 mM potassium chloride, 4.3 mM sodium phosphate, 1.4 mM potassium dihydrogen phosphate, 0.5% Tween-20) containing 5% skim milk for 2 h at RT and hybridized with the appropriate primary antibody (anti-EGFR (pY1068), anti-EGFR, anti-Ras, anti-c-Raf (pS338), anti-MEK 1/2 (pS217/221), anti-MAPK p44/42 (pT202/204), anti-p90RSK (pS380), anti-cytochrome c, anti-GAPDH (Cell Signaling Technology Inc., Danvers, MA, USA)) for overnight at 4°C. Protein bands were visualized by enhanced chemiluminescent (ECL) detection solution (Pierce, Rockford, IL, USA) after hybridization for 1 h with the horseradish peroxidase (HRP)-conjugated secondary antibody from rabbit or mouse (Cell Signaling Technology Inc.).

### Immunofluorescence of the phosphorylated EGFR

HeLa cells were incubated on cover glass-bottom dishes (SPL Lifesciences, Gyeonggi, Korea) in DMEM with high-glucose containing FBS (10%) and penicillin-streptomycin. The cells were fixed with 4% formaldehyde (Junsei Chemical Ltd., Japan) for 15 min at RT and then blocked for 1 h in 5% normal serum based on the host primary antibody. After removing the blocking buffer, cells were incubated with 0.1 μg/mL of anti-EGFR (pY1068) overnight at 4°C and then washed 3 times in cold PBS followed by incubation for 1 h with 0.1 μg/mL of anti-rabbit IgG (H + L), and F(ab') fragment (Alexa Fluor 488 conjugate) (Cell Signaling Technology Inc.). After washing, the stained cells were mounted with Prolong Gold Antifade Reagent (Invitrogen, Eugene, OR, USA) and then observed under a Nikon ECLIPS 50i microscope equipped with a charged-coupled device (CDD) camera (Nikon, Tokyo, Japan). Images were captured and processed with High-Content Analysis Software (Cambridge Healthtech Institute, Needham, MA, USA).

### Statistical analysis

The statistical significance of the differences between the values of compound-treated and non-treated groups was determined by GraphPad Prism 5.0. The results are expressed as mean values ± standard deviations of the mean (SDM). Every untreated control group and treated group was measured in differences by *t*-*tests*. (*p* < 0.05 was considered significant). The experiments were performed in triplicates and at least three times each. In case of no error bar in the graph, the variation of values is infinitesimal and thus, the bars are hidden behind.

## Competing interests

The authors declare that they have no competing interests.

## Authors’ contributions

MJ, SJ, BW, CY and GD participated in the study design, data collection, and data analysis and drafted the manuscript. MJ, SJ and GD participated in the data analysis and drafted and revised the manuscript. All authors read and approved the final manuscript.
